# Diffusion-Weighted MRI of Breast Lesions: A Five-Case Series Demonstrating Imaging Patterns and Diagnostic Pitfalls

**DOI:** 10.7759/cureus.118733

**Published:** 2026-10-28

**Authors:** Preeti Yadav, Arindam Mukherjee, Riya Samanta

**Affiliations:** 1 Radiology, World College of Medical Sciences, Jhajjar, IND; 2 Radiology, BLK Super Speciality Hospital, New Delhi, IND; 3 Radiodiagnosis, Lady Hardinge Medical College, New Delhi, IND

**Keywords:** apparent diffusion coefficient, breast lesions, breast mri, diffusion restriction, diffusion-weighted imaging, fibroadenoma, seroma

## Abstract

Diffusion-weighted imaging (DWI) is an adjunct MRI sequence that helps characterize breast lesions by providing valuable information about tissue microstructure based on the in vivo motion of water molecules. Malignant lesions typically restrict the free diffusion of water molecules, appearing bright on high b-value images and dark (with a low apparent diffusion coefficient (ADC)) on the ADC map. Some benign lesions can show similar DWI findings, leading to diagnostic challenges when DWI is used as an independent sequence for lesion characterization. We report five breast MRI cases demonstrating varied diffusion patterns and the pitfalls encountered during interpretation. The cases include invasive ductal carcinoma with regional axillary nodal disease, metaplastic carcinoma, fibroadenoma without true diffusion restriction, fibroadenoma with true diffusion restriction, and a post-procedural seroma. Both malignant lesions showed restricted diffusion. Among the two pathologically proven fibroadenomas, one showed T2 shine-through, whereas the other demonstrated true diffusion restriction. The post-procedural lesion showed facilitated diffusion despite thin rim post-contrast enhancement, suggestive of a seroma. These cases demonstrate the overlap in diffusion findings between benign and malignant breast lesions, emphasizing the importance of interpreting diffusion images in conjunction with ADC maps, lesion morphology, enhancement patterns, and clinical history.

## Introduction

Conventional breast MRI provides valuable morphological as well as enhancement-related information for characterization of breast lesions. Diffusion-weighted imaging (DWI) further adds functional information by providing insights into water mobility within tissues. The apparent diffusion coefficient (ADC), obtained from images acquired at different b-values, provides a quantitative measure of tissue diffusivity that is directly influenced by cellularity and tissue composition [1-3]. ADC measurements may be affected by factors such as region-of-interest (ROI) placement and lesion heterogeneity, including necrosis, cystic changes, and hemorrhage [4]. Malignant breast lesions generally demonstrate diffusion restriction due to increased cellularity and reduced extracellular space. However, these findings are not exclusive to malignant lesions [2].

Some benign lesions may produce misleading diffusion findings, leading to diagnostic difficulties. Most fibroadenomas show T2 shine-through, but some may show true diffusion restriction due to their histological composition [3]. In the post-procedural and lactating breast, DWI may provide useful complementary information for differentiating an abscess from a sterile post-procedural seroma. Physiological changes associated with lactation may also complicate MRI interpretation [5-7]. We present five illustrative breast MRI cases demonstrating contrasting diffusion patterns and potential diagnostic pitfalls in breast lesion evaluation.

## Case presentation

We present five illustrative cases of breast lesions evaluated at the World College of Medical Sciences and Research Hospital, Jhajjar, Haryana, India, between July 2025 and May 2026. The cases were purposively selected to highlight contrasting DWI patterns and potential diagnostic pitfalls rather than to represent consecutive patient enrolment. All patients underwent breast MRI on a 3-T Siemens MRI system (Siemens Healthineers, Erlangen, Germany) using appropriate breast coils. The protocol included T1-weighted, T2-weighted, and short tau inversion recovery (STIR) sequences, along with DWI using single-shot echo-planar imaging at b-values of 50 and 800 s/mm². Contrast-enhanced imaging was performed in selected cases. ADC maps were generated from the DWI data. For each lesion, the author manually placed a single ROI of approximately 5 mm² within the solid component, avoiding cystic and necrotic areas and preferentially excluding noisy voxels. The mean ADC value within the ROI was recorded using a consistent approach across all five cases.

Case 1: Invasive ductal carcinoma with axillary nodal disease

A 54-year-old nulliparous postmenopausal female presented with a painless lump in the left breast of one month’s duration, associated with mild nipple retraction without any nipple discharge. She reported no fever, significant weight loss, or comorbidities. On clinical examination, a firm, non-tender, poorly mobile lump was palpable in the left breast, along with a firm, enlarged left axillary lymph node. T2-weighted breast MRI sequences showed an irregular, mildly hyperintense mass in the inner quadrant of the left breast, with thickening of the overlying skin and nipple-areolar complex. A large, heterogeneous left axillary nodal mass was also present. On DWI, the primary lesion was markedly hyperintense, with corresponding hypointensity on the ADC map, suggestive of true diffusion restriction (Figure 1). The ADC value was 0.77 × 10⁻³ mm²/s. Similar diffusion restriction was also observed in the enlarged axillary nodal mass.

**Figure 1:**
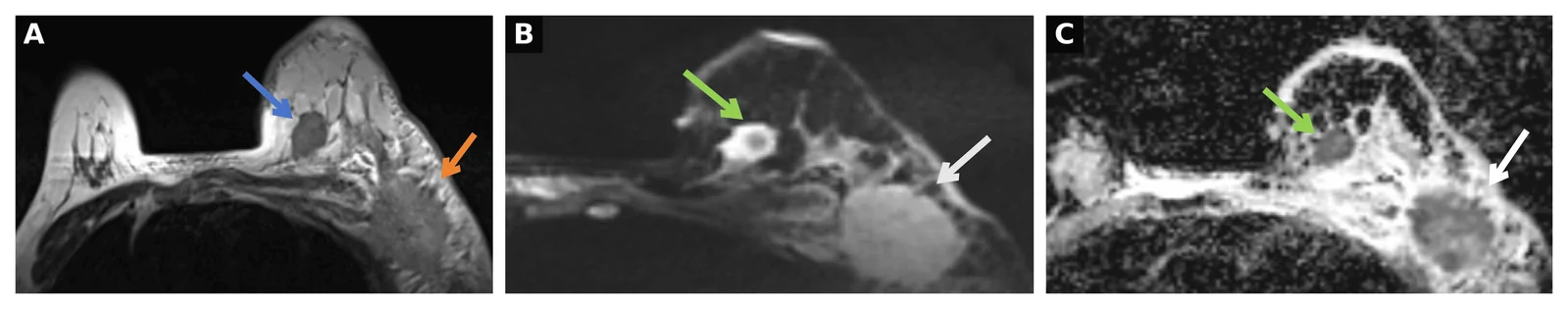
Findings in invasive ductal carcinoma with axillary nodal disease (A) Axial T2-weighted image shows an irregular, mildly hyperintense mass in the inner quadrant of the left breast (blue arrow) with nipple-areolar complex thickening and a large left axillary nodal mass (orange arrow). (B, C) Diffusion-weighted imaging demonstrates hyperintense signal in the primary breast lesion(Green arrows) and axillary nodal mass (white arrows), with corresponding low signal intensity on apparent diffusion coefficient maps, suggestive of true diffusion restriction

An irregular mass with skin and nipple-areolar involvement and marked diffusion restriction pointed to an advanced malignancy with regional nodal spread. Pathology confirmed invasive ductal carcinoma with axillary lymph node involvement.

Diagnostic Relevance

Restricted diffusion in the axillary nodal mass provided additional functional information supporting regional nodal involvement.

Case 2: Metaplastic carcinoma of the breast

A 48-year-old female presented with rapid enlargement of the right breast over a period of three months, associated with a large, painless lump. She also reported anorexia and a 7-kg weight loss over the same period. She had a family history of breast carcinoma in her maternal aunt. On examination, a large, firm, non-tender, non-mobile mass was palpable in the right breast. MRI showed a large, lobulated mass with heterogeneous T2 signal involving the outer quadrant and extending into the inner quadrant of the right breast, with posterior extension toward the chest wall. DWI demonstrated multifocal peripheral areas of high signal with corresponding low signal on the ADC map, indicating heterogeneous areas of diffusion restriction (Figure 2). The ADC measured in the most restricted component was 0.72 × 10⁻³ mm²/s.

**Figure 2:**
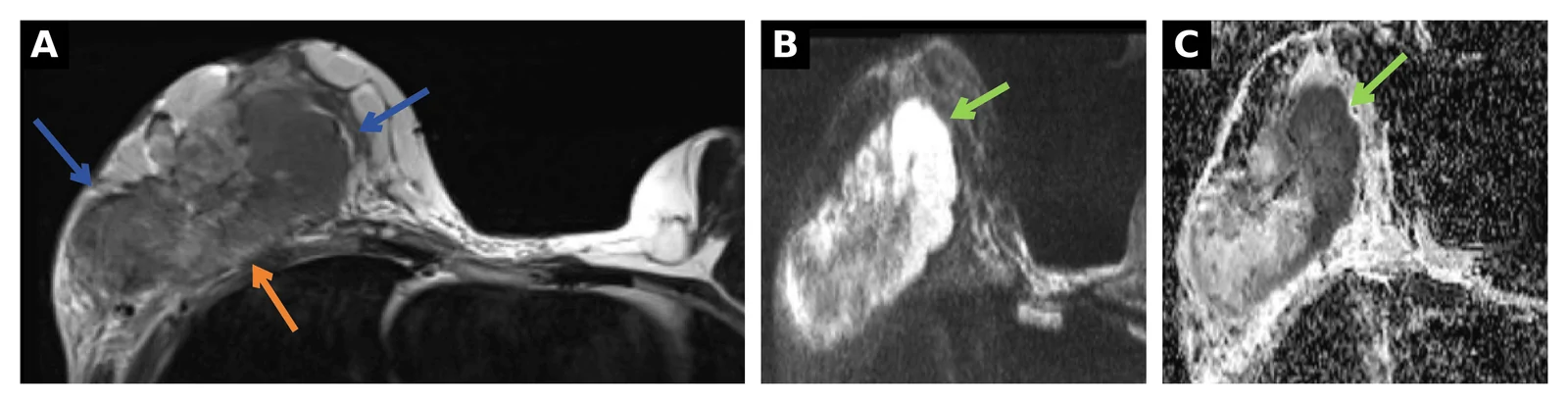
Findings in metaplastic carcinoma of the right breast (A) Axial T2-weighted image depicts a large lobulated mass (blue arrows) showing heterogeneous internal signal characteristics and posterior extension toward the chest wall, contacting the pectoralis muscle (orange arrow). (B, C) Axial diffusion-weighted and corresponding apparent diffusion coefficient images demonstrate heterogeneous, patchy areas of diffusion restriction within the mass (green arrows)

The large lobulated mass, extensive breast involvement, nipple retraction, posterior extension toward the chest wall, and areas of marked diffusion restriction raised concerns for an aggressive malignancy. Histopathological examination confirmed metaplastic carcinoma.

Diagnostic Relevance

This case highlights the importance of selecting representative viable solid components for ADC measurement in heterogeneous breast lesions.

Case 3: Fibroadenoma without true diffusion restriction

A 21-year-old woman presented with a painless palpable lump in the right breast for 18 months. Examination showed a well-defined, firm, mobile, non-tender retroaerolar lump. She had no fever, weight loss, or nipple discharge. The patient expressed anxiety because of a history of surgery for a similar lesion in the contralateral breast four years earlier. Although ultrasonography was offered, the patient preferred MRI for further evaluation of the current lump. Documentation regarding the nature and histopathology of the previously operated lesion was unavailable. MRI showed a well-circumscribed, oval, mildly T2 hyperintense lesion in the retroaerolar region of the right breast without architectural distortion, ductal dilatation, or axillary lymphadenopathy. The lesion showed uniformly high signal intensity on diffusion-weighted images and ADC map, consistent with T2 shine-through rather than true restricted diffusion (Figure 3). The ADC value was 1.56 × 10⁻³ mm²/s.

**Figure 3:**
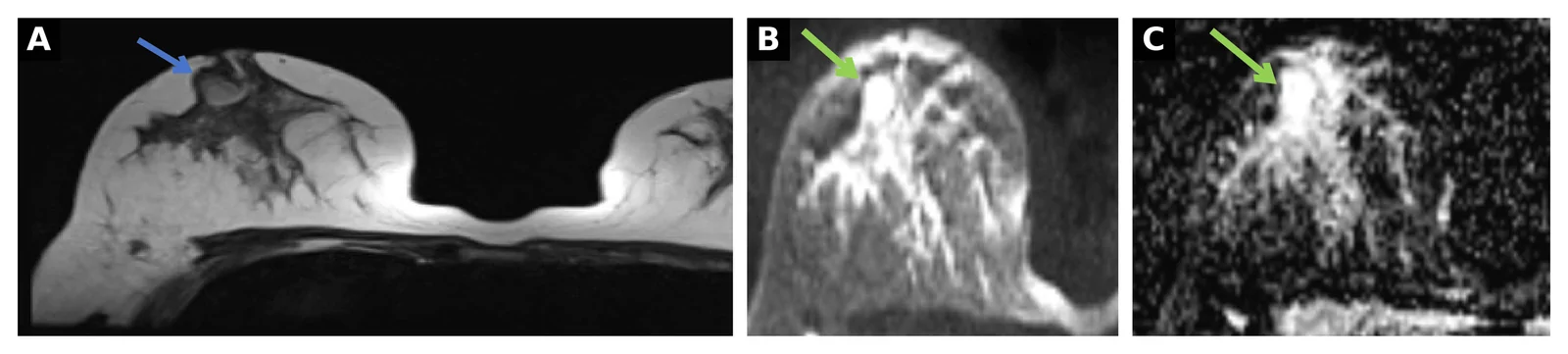
Findings in fibroadenoma of the right breast (A) Axial T2-weighted image reveals a well-circumscribed, oval, hyperintense lesion (blue arrow) in the retroaerolar region of the right breast. (B, C) Axial diffusion-weighted and corresponding apparent diffusion coefficient images show high signal intensity within the lesion (green arrows), consistent with T2 shine-through

Histopathological examination confirmed fibroadenoma.

Diagnostic Relevance

ADC map correlation is essential to distinguish T2 shine-through from true diffusion restriction in lesions showing high signal intensity on DWI.

Case 4: Fibroadenoma with true diffusion restriction

A 19-year-old female presented with a lump in the left breast that she had noticed 10 months earlier. Examination revealed a well-defined, firm, mobile, non-tender lump in the medial aspect of the left breast. There were no associated comorbidities or family history of breast lumps. Breast ultrasonography revealed an oval, circumscribed, heterogeneously hypoechoic lesion with prominent internal vascularity in the inner quadrant of the left breast. Breast MRI was requested by the clinician for further evaluation given the lesion’s sonographic features, particularly its internal vascularity. Breast MRI revealed a well-defined, oval, T2-hyperintense lesion with circumscribed margins in the inner quadrant of the left breast, with no ductal dilatation, skin thickening, or axillary lymphadenopathy. DWI showed intralesional high signal intensity with corresponding low signal intensity on the ADC map, indicating true diffusion restriction (Figure 4). The ADC value was 0.9 × 10⁻³ mm²/s.

**Figure 4:**
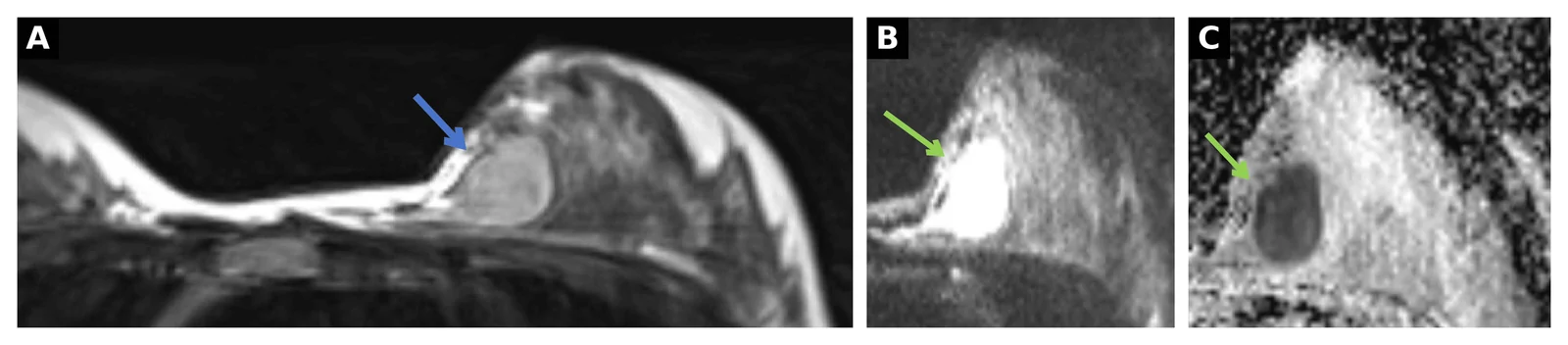
Findings of fibroadenoma with diffusion restriction (A) Axial T2-weighted image shows a well-defined, oval, hyperintense lesion with circumscribed margins (blue arrow) in the medial quadrant of the left breast. (B, C) Diffusion-weighted images exhibit high signal intensity with corresponding low signal intensity on apparent diffusion coefficient images (green arrow), representing true diffusion restriction

The diffusion characteristics were discordant with the otherwise benign-appearing lesion morphology. Histopathology confirmed a fibroadenoma.

Diagnostic Relevance

This case demonstrates that true diffusion restriction is not specific to malignant lesions and may also occur in some benign lesions, such as highly cellular fibroadenomas.

Case 5: Post-procedural seroma in a lactating woman

A 29-year-old lactating female presented with left breast swelling of one month’s duration, associated with a mild, intermittent aching sensation. Three months earlier, she had been diagnosed with lactational mastitis and a breast abscess, for which she underwent incision and drainage. Clinical examination showed a firm, smooth, mildly tender swelling, with a visible scar at the site of the previous procedure. She reported no fever, chills, weight loss, or anorexia. Ultrasonography revealed a well-defined, oval, thin-walled fluid collection with posterior acoustic enhancement and low-level internal echoes at the site of the previous procedure in the retroareolar region of the left breast, with no internal vascularity. The treating clinician suspected recurrent or residual mastitis or a breast abscess; therefore, breast MRI was requested for further evaluation.

Breast MRI demonstrated a well-defined, round, circumscribed lesion in the left breast, which was T2-hyperintense with a thin, smooth hypointense peripheral rim. The lesion showed thin, smooth peripheral rim enhancement after administration of gadolinium. The lesion was hypointense on DWI and hyperintense on the ADC map, suggestive of facilitated diffusion (Figure 5). The lesion was predominantly cystic, with a thin peripheral rim and no identifiable solid component. Therefore, quantitative ADC measurement was not performed, as the measurement protocol was intended for the solid component of the lesion.

**Figure 5:**
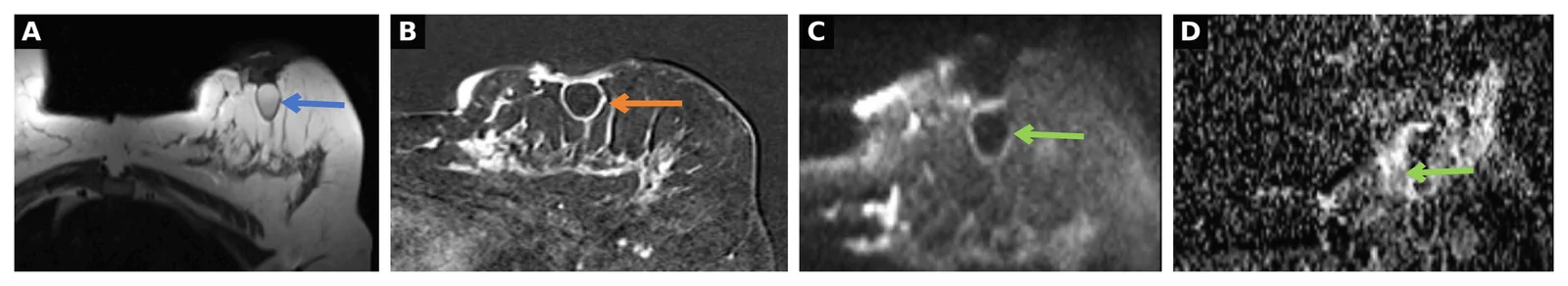
Findings of a post-procedural seroma in a lactating woman (A) Axial T2-weighted image depicts a well-defined, round, hyperintense lesion with a thin, smooth peripheral hypointense rim (blue arrow) in the left breast. (B) Post-gadolinium T1-weighted image demonstrates smooth peripheral rim enhancement (orange arrow). (C, D). The lesion shows hypointense signal on axial diffusion-weighted images (green arrow) and hyperintense signal on apparent diffusion coefficient images (green arrow), indicating facilitated diffusion

In view of the patient's history of previous intervention, lesion morphology, and diffusion characteristics, a post-procedural seroma was favored. Histopathology showed fibro-collagenous and granulation tissue with mild chronic inflammatory infiltrate containing proteinaceous serous material, with no atypia or malignancy, consistent with a post-procedural seroma.

Diagnostic Relevance

The combined use of morphology and diffusion findings helped to distinguish a post-procedural seroma from recurrent abscess, potentially avoiding unnecessary repeat drainage.

## Discussion

DWI provides functional information about the microscopic movement of water molecules within tissues, thereby complementing the morphological and enhancement-related information obtained from conventional MRI. Malignant breast lesions frequently exhibit increased cellularity and reduced extracellular space, resulting in restricted water mobility and increased signal intensity on high b-value DWI, with corresponding low signal intensity on ADC maps [2,3,8]. However, there is considerable overlap between the diffusion characteristics of benign and malignant lesions, limiting the reliability of DWI as an independent diagnostic criterion [9,10]. A multiparametric and integrated diagnostic approach combining diffusion characteristics with lesion morphology, enhancement patterns, clinical findings, and histopathological correlation, whenever indicated, is therefore essential for appropriate lesion characterization.

In the present series, the invasive ductal carcinoma demonstrated marked diffusion restriction, consistent with its irregular morphology, skin and nipple-areolar thickening, and associated axillary nodal disease. The axillary mass also demonstrated restricted diffusion, suggesting nodal involvement, which was confirmed by histopathology, highlighting the complementary role of DWI in evaluating the primary tumor and regional nodal involvement.

The metaplastic carcinoma revealed heterogeneous or patchy diffusion restriction. Metaplastic carcinomas may present as large masses with variable epithelial and mesenchymal components, and their imaging appearance may vary according to the internal composition of the tumor [11,12]. Differences in cellularity between viable tumor and areas of necrosis, cystic degeneration, or hemorrhage may explain the non-uniform diffusion pattern. ADC measurements should therefore be obtained from representative viable solid components.

The two fibroadenomas included in the case series demonstrated contrasting diffusion patterns. In Case 3, the lesion was hyperintense on high b-value diffusion images as well as on the ADC map, indicating T2 shine-through rather than true restriction. Because diffusion images retain substantial T2 weighting, intrinsically T2-hyperintense lesions may appear bright on DWI despite unrestricted water movement [1,13]. Evaluation of the corresponding ADC map is therefore critical for determining whether apparent DWI hyperintensity represents genuine restriction.

Case 4 demonstrated true diffusion restriction in a histopathologically benign fibroadenoma. Fibroadenomas have variable epithelial and stromal cellularity, which can influence their diffusion characteristics [3,14]. A relatively cellular fibroadenoma may have reduced extracellular space and consequently lower ADC values. ADC measurements are also influenced by ROI placement and the selection of different lesion components, which may contribute to variability between studies [15].

The fifth case describes a lactating woman who had previously undergone incision and drainage for a breast abscess. MRI demonstrated a well-defined, T2-hyperintense collection showing peripheral rim enhancement without convincing diffusion restriction. The peripheral rim enhancement may reflect vascularized granulation tissue surrounding the collection. The absence of convincing diffusion restriction, together with the history of prior intervention, favored a post-procedural seroma, which was subsequently confirmed by histopathology. DWI may provide useful complementary information for evaluating post-procedural collections in the lactating breast, where physiological changes can complicate MRI interpretation [5,7].

A limitation of this case series is the use of a single ROI per lesion for ADC assessment, which may not fully capture intralesional heterogeneity in diffusion characteristics. The findings should therefore be interpreted in light of the descriptive nature of the case series and its limited sample size.

## Conclusions

DWI is a useful adjunct to conventional breast MRI but cannot reliably distinguish benign from malignant lesions when used in isolation. This five-case series illustrates the diversity of diffusion patterns and associated diagnostic pitfalls, including the overlap in diffusion characteristics between benign and malignant lesions. Integrating diffusion findings with lesion morphology, enhancement characteristics, and clinical context, alongside histopathological correlation where indicated, facilitates a comprehensive diagnostic assessment.

## References

[1] Brandão AC, Lehman CD, Partridge SC (2013). Breast magnetic resonance imaging: diffusion-weighted imaging. Magn Reson Imaging Clin N Am.

[2] Woodhams R, Ramadan S, Stanwell P (2011). Diffusion-weighted imaging of the breast: principles and clinical applications. Radiographics.

[3] Baltzer P, Mann RM, Iima M (2020). Diffusion-weighted imaging of the breast-a consensus and mission statement from the EUSOBI International Breast Diffusion-Weighted Imaging working group. Eur Radiol.

[4] Partridge SC, Nissan N, Rahbar H, Kitsch AE, Sigmund EE (2017). Diffusion-weighted breast MRI: Clinical applications and emerging techniques. J Magn Reson Imaging.

[5] Talele AC, Slanetz PJ, Edmister WB, Yeh ED, Kopans DB (2003). The lactating breast: MRI findings and literature review. Breast J.

[6] Nissan N, Bauer E, Moss Massasa EE, Sklair-Levy M (2022). Breast MRI during pregnancy and lactation: clinical challenges and technical advances. Insights Imaging.

[7] Xu K, Chung M, Hayward JH, Kelil T, Lee AY, Ray KM (2024). MRI of the lactating breast. Radiographics.

[8] de Bazelaire C, Pluvinage A, Chapelier M (2010). Diffusion-weighted MR imaging of the breast (Article in French). J Radiol.

[9] Baxter GC, Graves MJ, Gilbert FJ, Patterson AJ (2019). A meta-analysis of the diagnostic performance of diffusion MRI for breast lesion characterization. Radiology.

[10] Partridge SC, DeMartini WB, Kurland BF, Eby PR, White SW, Lehman CD (2009). Quantitative diffusion-weighted imaging as an adjunct to conventional breast MRI for improved positive predictive value. AJR Am J Roentgenol.

[11] Jia Y, He C, Liu L, Sun L, Chen Y, Luo Y, Yu T (2019). A retrospective study of the imaging and pathological features of metaplastic breast carcinoma and review of the literature. Med Sci Monit.

[12] Bian T, Lin Q, Wu Z, Cui C, Qi C, Li L, Su X (2016). Metaplastic carcinoma of the breast: Imaging and pathological features. Oncol Lett.

[13] Thomassin-Naggara I, De Bazelaire C, Chopier J, Bazot M, Marsault C, Trop I (2013). Diffusion-weighted MR imaging of the breast: advantages and pitfalls. Eur J Radiol.

[14] Surov A, Meyer HJ, Wienke A (2019). Can apparent diffusion coefficient (ADC) distinguish breast cancer from benign breast findings? A meta-analysis based on 13 847 lesions. BMC Cancer.

[15] Bickel H, Pinker K, Polanec S (2017). Diffusion-weighted imaging of breast lesions: Region-of-interest placement and different ADC parameters influence apparent diffusion coefficient values. Eur Radiol.

